# Bacterial Community Composition in Oligosaline Lake Bosten: Low Overlap of *Betaproteobacteria* and *Bacteroidetes* with Freshwater Ecosystems

**DOI:** 10.1264/jsme2.ME14177

**Published:** 2015-05-15

**Authors:** Xiangming Tang, Guijuan Xie, Keqiang Shao, Jiangyu Dai, Yuangao Chen, Qiujin Xu, Guang Gao

**Affiliations:** 1 State Key Laboratory of Lake Science and Environment, Nanjing Institute of Geography and Limnology, Chinese Academy of SciencesNanjing 210008China; 2 State Key Laboratory of Hydrology-Water Resources and Hydraulic Engineering, Nanjing Hydraulic Research InstituteNanjing 210029China; 3 Chinese Research Academy of Environmental SciencesBeijing 100012China

**Keywords:** Lake Bosten, oligosaline, diversity, bacterial community composition, seasonal dynamics, salinity

## Abstract

Oligosaline lakes in arid regions provide indispensable water resources for humans; however, information on the bacterial community composition (BCC) of this ecosystem is limited. In the present study, we explored seasonal and vertical variations in BCC in Lake Bosten, a unique oligosaline lake (1.2‰ salinity) in arid, northwestern China, using denaturing gradient gel electrophoresis and 16S rRNA gene sequencing. We obtained 544 clones and 98 operational taxonomic units (OTUs) from six clone libraries. The top 10 OTUs represented 59.4% of the entire bacterial community. *Betaproteobacteria* (22.1%), *Gammaproteobacteria* (19.9%), *Bacteroidetes* (18.8%), and *Firmicutes* (11.4%) dominated in Lake Bosten. Although seasonal variations were recorded in BCC, the vertical changes observed were not significant. Water temperature and salinity were the most important factors shaping the dynamics of BCC. A low degree of overlap was observed in BCC between Lake Bosten and freshwater ecosystems, especially for *Betaproteobacteria* and *Bacteroidetes*. An RDP seqmatch analysis showed that 169 sequences (31%) were novel bacterial sequences (<97% similarity to the closest sequences in GenBank), which suggested that specific indigenous bacteria inhabit this oligosaline environment. Our results support bacterial endemicity being more common than previously considered, particularly in oligosaline lakes. An analysis of these communities may reveal how bacteria respond to increases in salinity and nutrients in the early stage of salinization and eutrophication.

Arid and semiarid regions account for almost one-third of the world’s and half of China’s land area, respectively. Lakes in these regions provide sparse, but valuable water resources for fragile environments and humans. However, significant decreases have occurred in the surface areas of most main inland lakes in arid central Asia in the past 30 years due to regional climate changes and recent anthropogenic activities (3). Lakes located in arid or semi-arid regions have been described as early indicators of both regional and global environmental changes, and bacteria are thought to be a sensitive sentinel of these changes (1, 55).

Heterotrophic bacteria are a major constituent of aquatic ecosystems, in which they play a prominent role in the breakdown of organic compounds, thereby contributing to the biodegradation of pollutants (2, 7, 13, 34). Determining bacterial community composition (BCC) and responses to the changing environment is one of the necessary steps in understanding aquatic microbial ecology (38). Although many studies have focused on bacterial diversity and community composition in saline and hypersaline systems (10, 18, 37) as well as freshwater habitats (12, 34, 52, 61), BCC in oligosaline lakes located in arid and semiarid regions has not been examined in as much detail (15).

Lake Bosten is the largest lake in Xinjiang Province, northwest China. The drainage basin of Lake Bosten lies in the center of the Eurasian continent, in which the combination of sunlight and heat, mean annual precipitation of 64 mm, and mean annual evaporation of 1,881 mm produce an inland desert climate (3). In the past 50 years, the salinity of Lake Bosten has increased from 0.38 g L^−1^ to 1.46 g L^−1^, and, accordingly, nutrient levels have also increased from oligotrophic to mesotrophic levels (58). While horizontal variations in BCC in the surface water and sediment of Lake Bosten have recently been reported (9, 50), the seasonal and vertical dynamics of bacterial communities are still poorly understood. Furthermore, the responses of detailed bacterial phylotypes to increased salinity and nutrition levels in oligosaline and mesotrophic lakes have not yet been elucidated in detail.

In the present study, we used 16S rRNA gene-based denaturing gradient gel electrophoresis (DGGE) and sequencing of selected clone libraries to examine BCC in Lake Bosten at different stations and in different seasons. Our specific goals were to (i) characterize seasonal and vertical dynamics in BCC in Lake Bosten, (ii) identify the environmental and spatial factors shaping variations in BCC, and (iii) determine whether indigenous bacterial clusters inhabit this oligosaline lake compared with freshwater ecosystems. Since shifts in bacterial phylotypes may be rapid biological proxies of change, the results of this study will be particularly important for understanding the response patterns of BCC in the early stage of lake salinization and eutrophication.

## Materials and Methods

### Study area and sampling procedures

**Fig. 1:**
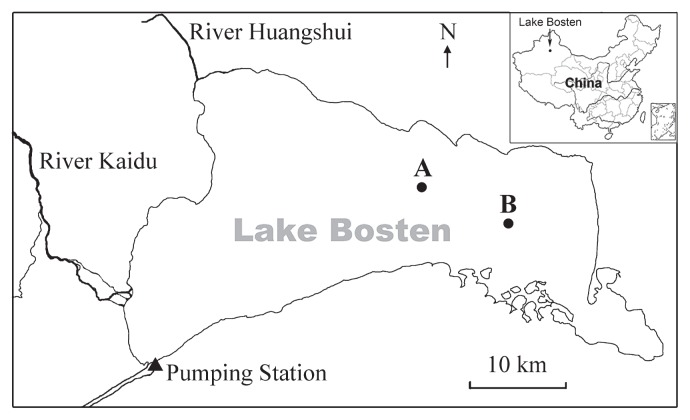
Sampling stations in Lake Bosten.

Lake Bosten (86°40′–87°26′ E and 41°56′–42°14′ N) is a meromictic lake with a surface area of approximately 950 km^2^ (1,046 m above sea level), a maximum depth of 16 m, and a mean depth of 7 m (Fig. 1). The maximum lake length is 57 km and maximum width is 31 km. River Kaidu, originating in the snow- and glacier-covered Tianshan mountains, is the main river feeding Lake Bosten. The average annual runoff is 34×10^8^ m^3^, which supplies 85% of the water volume of Lake Bosten (58). River Huangshui, another permanent river feeding Lake Bosten, has markedly higher concentrations of nutrients and salt than River Kaidu due to pollution from agricultural and industrial wastewater. Water from Lake Bosten is pumped by two pumping stations into artificial channels at its southwestern margin (Fig. 1).

To minimize the impact of hydrology on BCC, we selected two sampling stations far from River Kaidu and River Huangshui: Station A was located in the open lake and Station B was located 9.7 km southeast of Station A. The water depth of both stations was >12 m, and the mean water residence time was >970 d (19).

Water samples were collected at the two stations using a 5-L Schindler sampler on 23 August 2010 and 10 May 2011 at 4-m intervals from the surface (top 0.5 m) to a depth of 12 m. A subsample of water (250 mL) for the 16S rRNA gene analysis was collected on 0.2-μm pore-size polycarbonate filter (Millipore) *in situ* using a hand-driven vacuum pump. These filters were frozen at −80°C until DNA extraction was performed. Sub-samples (46 mL) were transferred into autoclaved tubes (Greiner Bio-one GmbH, Germany) containing 4 mL of prefiltered (pore size, 0.2 μm) glutaraldehyde (final concentration 2% [v/v]). Samples were then stored in a refrigerator at 4°C until slides were prepared for enumeration of bacterial abundance. The remaining water samples were transported to the laboratory within 4 h for immediate chemical analysis.

### Measurement of environmental parameters and enumeration of bacteria

Water depth and Secchi transparency depth were measured on location using a water depth gauge (Uwitec, Austria) and Secchi disk, respectively. Water temperature, dissolved oxygen (DO), pH, electrical conductivity (EC), total dissolved solids (TDS), and salinity were measured at 2-m intervals in the field using a multiparameter water quality sonde (YSI 6600V2, USA). Chemical analyses of water samples comprising total nitrogen (TN), ammonium (NH_4_-N), nitrate (NO_3_-N), total phosphorus (TP), chloride (Cl^−^), sulfate (SO_4_^2−^), dissolved organic carbon (DOC), and chlorophyll *a* (Chl *a*) were measured in the laboratory according to standard methods (19).

The abundance of bacteria in the water samples was determined by the 4′,6′-diamidino-2-phenylindole (DAPI)-combined epifluorescence direct counting method (22, 48).

### DNA extraction, PCR amplification, and DGGE analysis

Total DNA for filtered microorganisms was extracted according to Zhou *et al.* (60). Crude DNA extracts were purified using the E.Z.N.A. Cycle-Pure Kit (Omega Bio-Tek). To amplify the V3 region of the bacterial 16S rRNA gene, the primers 341f (5′-CCTACGGGAGGCAGCAG-3′) and 534r (5′-ATTACCGCGG CTGCTGG-3′) were used, with a 40-base-pair GC-clamp attached to the 5′ end of the forward primer (32). Polymerase chain reaction (PCR) amplification was performed in 50 μL reaction mixture containing 5 μL of 10×PCR buffer, 4 μL of MgCl_2_ (25 mM), 3 μL of deoxynucleotide triphosphates (dNTPs, 2.5 mM each), 1 μL of each primer (10 μM), 10–50 ng template DNA, and 0.3 μL of *Taq* polymerase (5 U μL^−1^ Fermentas).

PCR cycling was carried out in a thermocycler (Applied Biosystems Veriti Thermal Cycler) using a touchdown program: denaturation at 94°C for 5 min, 11 cycles of denaturation at 94°C for 1 min, annealing at 65°C for 1 min (temperature was decreased by 1°C every cycle until 55°C was reached), and extension at 72°C for 1 min. Nineteen additional cycles were carried out at an annealing temperature of 55°C, followed by a final extension at 72°C for 10 min.

Triplicate PCR products were mixed, then purified and concentrated using the E.Z.N.A. Cycle-Pure Kit. Samples containing approximately equal amounts of PCR amplicons and three prefabricated standard DNA markers were loaded onto 8% polyacrylamide gels (37.5:1, acrylamide/bisacrylamide) in 1×TAE buffer (40 mM Tris-acetate, pH 8.5, 1 mM Na_2_ EDTA) using a denaturing gradient ranging from 37% to 57% (the 100% denaturant contained 7 M urea and 40% formamide). Electrophoresis was performed at 100 V and 60°C for 16 h with a DGGE-2001 system (C.B.S. Scientific). Gels were stained with SYBR Green I solution (1:10,000 dilution, Amresco) for 30 min and destained in TAE buffer (pH 7.5) for 15 min. Images were acquired using an Omega 10 gel documentation system (Ultra-Lum Inc., USA). Gel images were analyzed with GelCompar II software (Applied Maths, Belgium). A matrix of relative band intensities and a binary matrix scored as present (1) or absent (0) were generated for subsequent analyses. Bands with a relative intensity <0.5% were discarded. A cluster analysis of DGGE profiles was performed using the Jaccard similarity index: *J*=100(*c*/[*a*+*b*−*c*]); where *a* is the number of bands of the lane A, *b* the number of bands of lane B, and *c* the number of bands common to lanes A and B.

### Clone library construction, diversity index, and phylogenetic analysis

Based on the results of the cluster analysis of DGGE profiles, DNA extracted from four samples collected at both Stations A and B at depths of 0.5 m and 12 m on 23 August 2010 (*i.e.*, Aug-A-0.5m, Aug-A-12m, Aug-B-0.5m, and Aug-B-12m) and two samples collected at Station A at the depths of 0.5 m and 12 m on 10 May 2011 (*i.e.*, May-A-0.5m and May-A-12m) were selected for construction of the 16S rRNA gene clone libraries. The general bacterial primers 8F (5′-AGAGTTTGATCMTGGCTCAG-3′) and 1492R (5′-GGT TACCTTGTTACGACTT-3′) were used in PCR amplification (33). The PCR products were purified immediately, and the 16S rRNA gene fragments were cloned into the pMD19-T simple vector (TaKaRa) following the manufacturer’s instructions. The randomly chosen clones were amplified directly from cells using the vector primers RV-M and M13-47 to determine the sizes of the inserts and exclude false positive clones.

Positive clones (100 per library) were sequenced on an automated DNA capillary sequencer (model 3730; Applied Biosystems) using the primer 8F and ABI Prism BigDye terminator sequencing kit v3.1 (Applied Biosystems). All partial 16S rDNA sequences were edited manually using the software BioEdit version 7.0.9 (16), aligned with ClustalW, and then grouped together based on sequence similarities. All assembled sequences were examined for chimerical artifacts using the Ribosomal Database Project II Chimera Check program (6) and UCHIME program (11). Twenty chimerical sequences and 36 chloroplast sequences were excluded from further analysis. Sequences with >97% similarity to each other were treated as a single operational taxonomic unit (OTU). The closest sequences were retrieved from the NCBI database (http://www.ncbi.nlm.nih.gov/blast/) using BLAST. Sequences were assigned to the genus level with >80% confidence using the “Classifier” program of the Ribosomal Database Project (RDP Release 10, http://rdp.cme.msu.edu/) to obtain preliminary phylogenetic affiliations (6).

The sequencing-based distribution of clones in different OTUs was used for each clone library to estimate the 16S rRNA gene library size and coverage. The diversity index, *i.e.*, the Chao1 richness estimator, reciprocal Simpson’s dominance index (RSI), and Shannon’s diversity index (*H*′) were calculated according to Chao *et al.* (5), Hill *et al.* (17), and Tang *et al.* (48) using SPADE (Species Prediction and Diversity Estimation; Chao and Shen, http://chao.stat.nthu.edu.tw/softwareCE.html).

Phylogenetic trees including the obtained OTUs, their closest relatives, and sequences in the 72 typical freshwater bacterial clusters (Table S1) (8, 12, 57, 61) were constructed using Molecular Evolutionary Genetics Analysis (MEGA) software v5.2 (45, 47). Evolutionary history was inferred using the Maximum Likelihood method based on the Jukes-Cantor model (14). The robustness of the tree topology was confirmed by Maximum Parsimony with 1,000 bootstrap replications.

### Statistical analysis

A canonical correspondence analysis (CCA) was used to examine the influence of the explanatory environmental variables on variations in the bacterial communities obtained from DGGE profiles. The 14 environmental variables tested were water depth, water temperature, pH, DO, EC, salinity, TDS, TN, NH_4_-N, NO_3_-N, Cl^−^, SO_4_^2−^, DOC, and Chl *a*. All environmental parameters were log (*x*+1) transformed and standardized. CCA was computed with the software CANOCO 4.5 using the linear species–environment relationship method because a detrended correspondence analysis (DCA) run on a DGGE profile matrix indicated that the length of the first axis was >3 (51). Environmental variables were identified by forward selection using a Monte Carlo test with 499 permutations.

In order to determine differences among the six selected communities, the constructed clone libraries were compared statistically with the *∫*-Libshuff program using the software Mothur v1.24.0 (41). The libraries were considered significantly different if the *P* value was <0.0017 (40, 44).

### Deposition of nucleotide sequence accession numbers

The partial bacterial 16S rRNA gene sequences determined in the present study were deposited in GenBank with the accession numbers JQ327161–JQ327704.

## Results

### Variations in physicochemical parameters and bacterial abundances

**Fig. 2:**
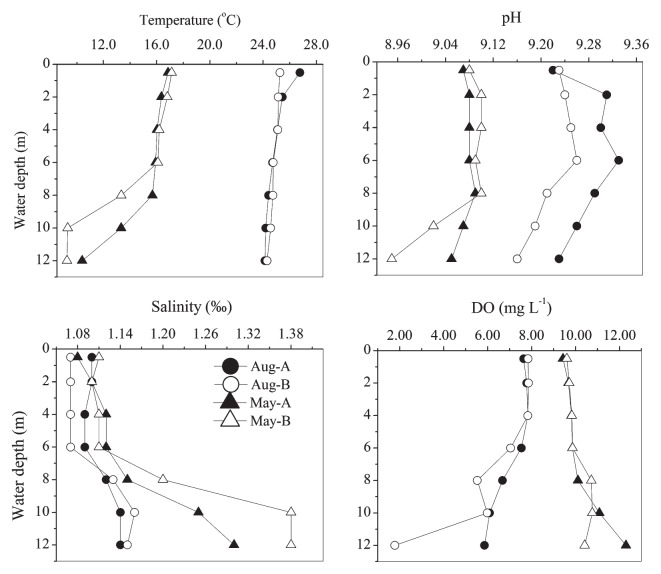
Vertical profiles of temperature, pH, salinity, and dissolved oxygen (DO) on 23 August 2010 and 10 May 2011, respectively.

In August, water temperature remained stable at approximately 25°C through different water depths at both stations. In contrast, water temperature in May peaked at approximately 17°C at the surface and then markedly declined below 8 m, reaching 9–10°C at the bottom (Fig. 2). Salinity remained stable at approximately 1.10‰ when water depth was above 6 m, but increased to 1.38‰ at a depth of 10 m in May at Station B. The concentration of DO was higher in May (≈10 mg L^−1^) that in August (≈7 mg L^−1^). The bottom water lacked oxygen (1.78 mg L^−1^) in August at Station B (Fig. 2). Bacterial abundances ranged from 0.49 to 3.86×10^6^ cells mL^−1^ (see Table S2 in the Supplement for other environmental parameters).

### Variations in BCC and related environmental factors

**Fig. 3:**
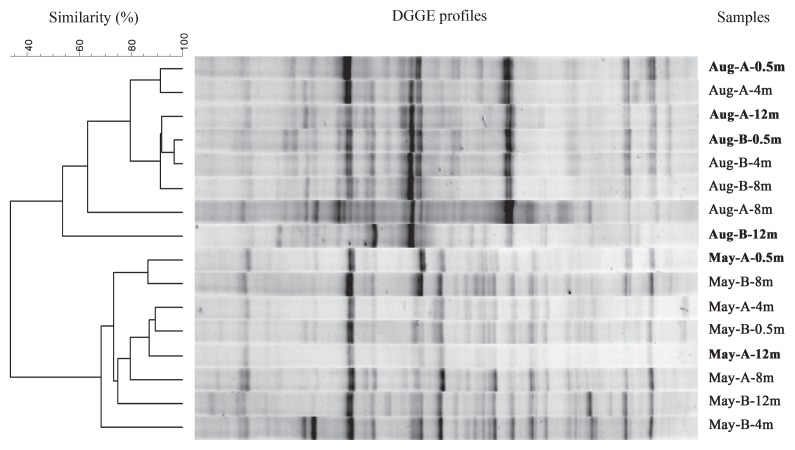
DGGE profiles of bacterial PCR-amplified 16S rRNA gene fragments obtained from samples collected from depths of 0.5 to 12 m at two stations in Lake Bosten on 23 August 2010 and 10 May 2011. The dendrogram was created by a cluster analysis of DGGE profiles obtained based on the unweighted pairgroup method with arithmetic averages (UPGMA) incorporating Pearson correlation’s coefficient of similarity. Samples selected for further 16S rRNA gene clone library analyses are indicated in bold.

The results of the DGGE analysis revealed the distinct separation of BCC between August and May (Fig. 3). Vertical variations in BCC were not notable in May samples, as shown by the dendrograms with similarity >60% among the DGGE profiles. In August samples, vertical variations in BCC were similarly indistinct, except for one at a depth of 12 m with a markedly lower concentration of DO (Fig. 2 and Fig. 3).

**Fig. 4:**
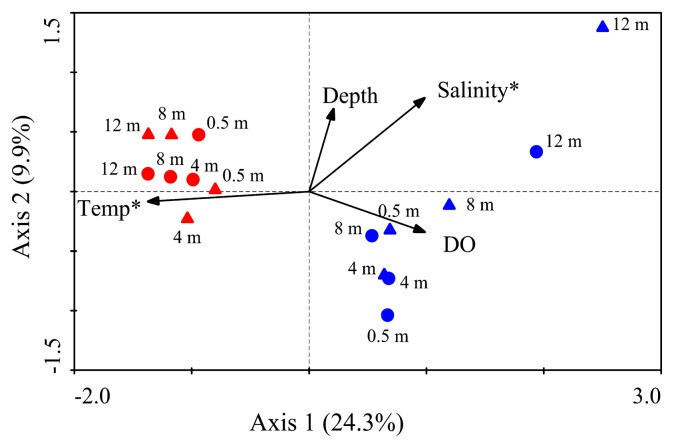
Canonical correspondence analysis biplots showed variations in BCC related to different environmental factors. The significant environmental factors identified by the Monte-Carlo test are marked with an asterisk. Circles represent samples from Station A, and up-triangles represent samples from Station B. Red symbols represent samples collected on 23 August 2010, and blue symbols represent samples collected on 10 May 2011. The numbers adjacent to the symbols represent water depth. Temp = water temperature. DO=dissolved oxygen.

The results of CCA indicated that four environmental factors, *i.e.*, water temperature, salinity, DO, and water depth, accounted for 34.2% of the variations in BCC on the two axes (Fig. 4). However, only water temperature (*p*=0.002, *F*=3.86) and salinity (*p*=0.038, *F*=1.55) contributed significantly to temporal and vertical variations in BCC.

### Significance test of differences between bacterial clone libraries

**Fig. 5:**
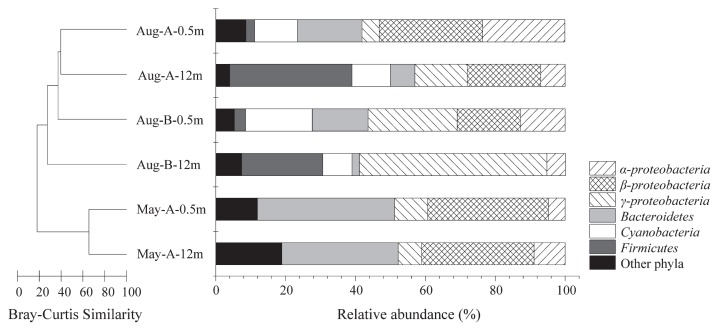
Relative abundance of dominant phyla to the total number of sequences detected in each sample. Clones affiliated with *Actinobacteria*, *Deinococcus-Thermus*, *Deltaproteobacteria*, *Planctomycetes*, and *Verrucomicrobia* are included in “Other phyla”. The dendrogram, as determined by Bray-Curtis similarity using OTU abundance data (>97% identity), illustrates the differences among the six bacterial communities sampled in Lake Bosten at two stations (Station A and Station B) and at two water depths (0.5 m and 12 m) in August 2010 and May 2011.

*∫*-Libshuff comparisons showed no significant differences between the two libraries in May (Table S3 in the Supplement), whereas libraries between May and August all differed significantly. In August, only library Aug-B-12m was considered significantly different from the other three libraries. These results were in accordance with the cluster analysis of the clone libraries (Fig. 5).

### Diversity patterns and phylogenetic composition of bacterial communities

Ninety-eight OTUs (97% cutoff) were obtained from 544 nonchimerical 16S rRNA sequences of the six selected clone libraries (Table 1). Coverage of the six libraries varied from 73.4% to 92.9%, with the number of OTUs in each library ranging from 18 to 40 (Table 1). The bacterial diversity of samples was lower in May than in August, as indicated from the lower OTUs, Chao 1, and Shannon’s diversity index (*H′*). Moreover, the diversity of the bottom sample (Aug-B-12m) with a low concentration of oxygen was markedly lower than that of the other samples from August (Table 1).

The phylogenetic analysis of the 544 sequences revealed that *Betaproteobacteria* was the most abundant bacteria, accounting for 22.1% of all sequences, followed in decreasing order by *Gammaproteobacteria* (19.9%), *Bacteroidetes* (18.8%), *Firmicutes* (11.4%), *Alphaproteobacteria* (10.1%), and *Cyanobacteria* (8.6%). Sequences affiliated with *Planctomycetes*, *Verrucomicrobia*, *Deinococcus-Thermus*, *Actinobacteria*, and *Deltaproteobacteria* were also detected at low frequencies; however, the relative abundance of bacterial phyla fluctuated with seasons and water depth. For example, the proportion of *Bacteroidetes* at Station A increased rapidly from 18.5% in August to 39.3% in May (Fig. 5). In August, the abundance of *Alphaproteobacteria* and *Bacteroidetes* was markedly higher in surface water than in bottom water, while *Firmicutes* presented an obvious, opposite trend. Furthermore, sample Aug-B-12m was more distinctive with other samples, which may be related to its low oxygen concentration. *Betaproteobacteria* was not detected in this sample; however, the proportion of *Gammaproteobacteria* accounted for 53.7% of all clones (Fig. 5).

**Fig. 6:**
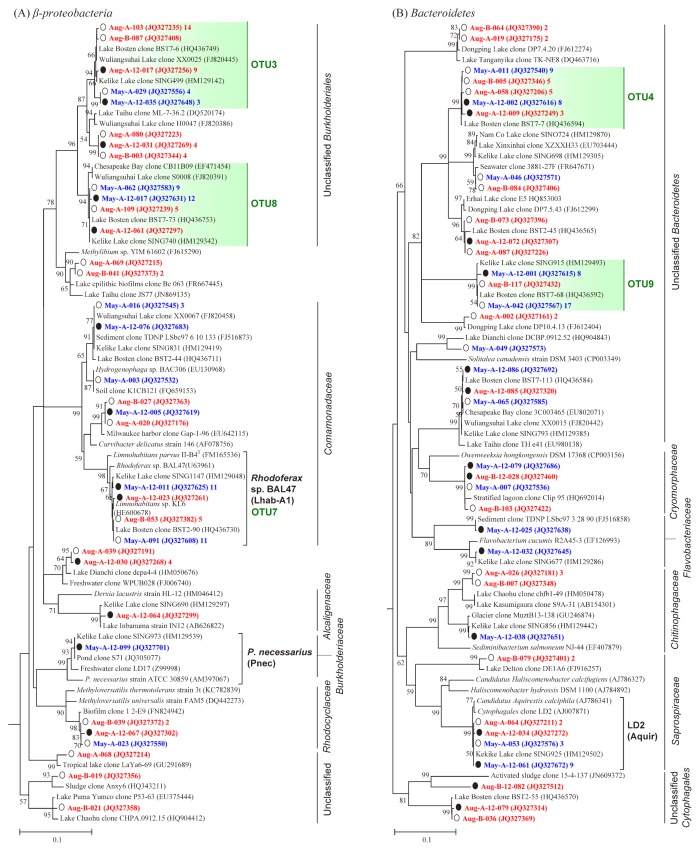
Phylogenetic trees of *Bacteroidetes* (A) and *Betaproteobacteria* (B) inferred by a Maximum Likelihood analysis of partial 16S rRNA gene sequences from six clone libraries in Lake Bosten. A bootstrap test with 1000 replicates was conducted, and only bootstrap values >50% are shown near nodes. Phylogenetic analyses were conducted in MEGA v5.2. Bar: 10% of estimated sequence divergence. Red clones were obtained in August 2010, and blue clones were obtained in May 2011. Only one representative clone from each library is shown for each OTU. The GenBank accession numbers are given in parentheses, followed by the number of clones within each representative clone. The most dominant 10 OTUs (Table 2) in the tree are shown in green. The open circles (○) before the clones represent surface water samples, and the dark filled circles (●) represent bottom water samples. Brackets following clone names indicate typical freshwater clusters previously reported by Crump & Hobbie (8), Eiler & Bertilsson (12), Wu *et al.* (57), and Zwart *et al.* (61). Names in brackets following the typical freshwater clusters were tribes or lineages named by Newton and coworkers (34).

Only 17.8% (97 out of 544) of all clones phylogenetically belonged to 13 already known freshwater clusters. LD12, A0904, *B. intermedius*, Liuu-9-115.2, *Rhodoferax* sp. BAL47, *Polynucleobacter necessarius* (*P. necessarius*), LD2, *Microcystis*, *Synechococcus* 6b, acII-B, STA2-30, LD19, and LiUU-9-218 (Fig. 6 and Fig. S1) were only detected among the 72 typical freshwater bacterial clusters (8, 12, 57, 61). LD12 as an *Alphaproteobacteria* cluster was the only group that contained OTUs from all six libraries.

In the present study, the top 10 abundant OTUs accounted for 50.6%–73.8% of all clones among the six libraries with an average of 59.4% (Table 2). The most abundant OTU (OTU1, Table 2) belonged to *Gammaproteobacteria* and accounted for 47% (51 out of 108) of all Gammaproteobacterial clones. It was most closely related (99.8% similarity; Table 2) to strain *Acinetobacter* sp. N40 (Fig. S1) from Lake Negra (salinity=32 mg L^−1^), Argentina, with high tolerance to UV radiation (36). OTU6 was assigned to the family *Chromatiaceae* (Fig. S1), which is representative of phototrophic purple bacteria. The most closely related strain to OTU6 was isolated from the Baltic Sea, which may be assigned to genus *Rheinheimera* (31).

*Firmicutes* displayed a relatively high proportion (11.4%) in the total community, with *Exiguobacterium* being the dominant genus (Fig. S1, Table 2). OTU2, accounting for 74% of the *Firmicutes*, is closely related (99.9% similarity) to strain *Exiguobacterium* sp. H1632, which was isolated from coastal seawater and has the ability to degrade cellulose. A previous study demonstrated that *Exiguobacterium* sp. was widely distributed in saline environments and presented high UV-B resistance (36).

OTU3, OTU7, and OTU8 belonged to *Betaproteobacteria* (Table 2). OTU7 was attributed to tribe Lhab-A1 (belonging to the genus *Limnohabitans*), one of the five subclusters of typical freshwater cluster *Rhodoferax* sp. BAL47 (34, 61). OTU3 and OTU8 belonged to unclassified *Burkholderiales* and do not currently have closely related strains (Fig. 6A). Among the top 10 OTUs, two came from *Bacteroidetes*: OTU4 and OTU9 (Table 2 and Fig. 6), which lacked cultivated representatives.

In total, 31% (169 out of 544) novel bacterial sequences (<97% similarity to the closest sequences in GenBank) were obtained in the six clone libraries based on the RDP seqmatch analysis conducted in August 2014. Of these, 70 sequences from *Proteobacteria* and 60 sequences from *Bacteroidetes* accounted for 25% and 59% of each phylum, respectively.

## Discussion

### Overlap of BCC in Lake Bosten with freshwater lake

One of the main aims of the present study was to determine whether indigenous bacterial clusters inhabited this oligosaline lake (salinity≈1.4 g L^−1^). Bacteria in Lake Bosten showed a weaker phylogenic relationship with those in freshwater habitats (Fig. 6 and Fig. S1). Our results were consistent with previous findings both in the surface water (50) and sediments (9) of Lake Bosten.

**Fig. 7:**
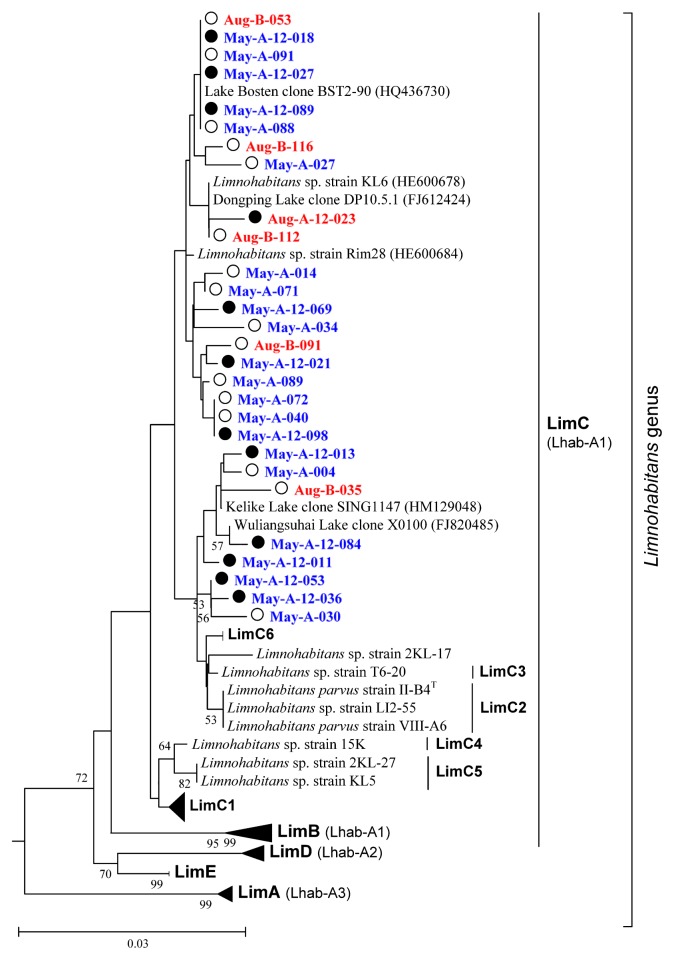
Phylogenetic tree of *Limnohabitans* clones obtained in Lake Bosten libraries, *Limnohabitans* spp. strains described by Kasalický *et al.* (20) and other environmental clones. A bootstrap test with 1000 replicates was conducted, and only bootstrap values >50% are shown near nodes. The scale bar corresponds to 3 base substitutions per 100 nucleotide positions. The tree was rooted by *Rhodoferax ferrireducens* T118. Red clones were obtained in August 2010, and blue clones were obtained in May 2011. The open circles (○) before the clones represent surface water samples, and the dark filled circles (●) represent bottom water samples. Bold names and names in brackets were lineages and tribes described by Kasalický *et al.* (20) and Newton *et al.* (34), respectively.

The low degree of overlap in BCC between Lake Bosten and freshwater ecosystems was particularly reflected in *Betaproteobacteria* and *Bacteroidetes*. *Betaproteobacteria*, distributed globally in freshwater lakes, are often the numerically dominating group inhabiting upper waters and organic particles (12, 34, 48, 61). In the present study, we found that *Betaproteobacteria* was the most abundant bacterial group, which is consistent with our previous findings (50). However, only two typical freshwater bacterial clusters were detected in Lake Bosten (Fig. 6). *Polynucleobacter*, the usually dominant freshwater cluster, was acquired with only one sequence. The genus *Limnohabitans* was the only cluster of typical freshwater bacteria ranging amongst the most important groups in Lake Bosten (Fig. 6A and Table 2). An in-depth phylogenetic analysis on the position of the Lake Bosten *Limnohabitans* in the phylogeny of *Limnohabitans* was performed (Fig. 7) under the detailed taxonomic framework for this genus presented by Kasalický *et al.* (20). Our results showed that only one lineage (*i.e.*, LimC) of *Limnohabitans* was present in Lake Bosten. Furthermore, these LimC clones only had two closely related strains (KL6 and Rim28) and none of them belonged to any of the six sublineages (LimC1–LimC6) proposed by Kasalický *et al.* (20). Due to the lack of isolated species, it is difficult to confirm whether these *Limnohabitans* clones represent a LimC sublineage that adapted to an oligosaline system.

Although *Bacteroidetes* formed the second largest phylum of the bacterial communities in Lake Bosten, they showed a weaker phylogenic relationship with those found in freshwater and marine environments because only one typical freshwater cluster (*i.e.*, LD2) was found with low abundance in this phylum (Fig. 6). The closest representatives of OTU4 were found in Lake Bosten, while the closest representative sequences of OTU9 were from Lake Kelike (59). These bacterial clusters appear to represent organisms adapted to oligosaline environments and local arid climatic conditions.

Bacteria recovered from Lake Bosten showed greater similarity with sequences obtained from lakes in arid northwestern China than from lakes in other places. For example, four out of the top 10 most abundant OTUs (Table 2) and 51.7% of all *Betaproteobacteria* clones (Fig. 6A and Fig. 7) were closely related to clones obtained in oligosaline Lake Kelike (900 km southeast of Lake Bosten in Qinhai Province; salinity≈1.1 g L^−1^) and/or Lake Wuliangsuhai (salinity≈2 g L^−1^) located in arid Inner Mongolia, northern China (46, 59). Moreover, the closest relatives of the most abundant two OTUs (OTU 1 and OTU 2) exhibited high UV resistance (Table 2), indicating the ecological niche adaptation of bacteria in the high solar irradiance Lake Bosten.

### Effects of environmental factors on BCC in Lake Bosten

In the present study, we found that some dominant OTUs, such as OTU1, OTU2, and OTU5 (Table 2), and *Cyanobacteria*, were only obtained in August libraries, suggesting a rapid seasonal variation in BCC in Lake Bosten. Moreover, *∫*-Libshuff comparisons showed that BCC between May and August all differed significantly (Table S3). CCA confirmed that water temperature was the most important factor shaping seasonal changes in BCC (Fig. 4). Water temperature as a main determiner triggers many biological processes and affects the succession of phytoplankton (54), thereby driving seasonal shifts in BCC in diverse lakes (26, 39, 49). Seasonal fluctuations in water temperature represent habitat heterogeneity and, thus, lead to idiosyncratic effects on the temporal dynamics of BCC (21, 42).

On a global scale, salinity has been identified as the major environmental determinant of microbial community composition (30). Within aquatic systems, salinity is often the dominant environmental factor controlling BCC from freshwater rivers to marine waters (25, 43) and in Tibetan lakes (28, 56, 59). Although salinity did not vary greatly in the present study (1.37–1.73 g L^−1^), it was found to be the second most important environmental factor, correlating with temporal and vertical variations in BCC (Fig. 4). The influence of salinity on structuring the spatial heterogeneity of BCC in surface water and sediment of Lake Bosten has been discussed elsewhere (9, 50). Salinity may directly affect the abundance, growth, and activity of bacterial communities and provide a physiological barrier for certain bacterial groups (4, 25, 29).

In Lake Bosten, most of the clusters in *Betaproteobacteria* and *Bacteroidetes* were distinct from previously named typical freshwater clusters (Fig. 6). However, the relatively high percentage of unclassified *Burkholderiales* (containing OTU3 and OTU8) and unclassified *Bacteroidetes* (containing OTU4 and OTU9) cannot solely be explained by the effect of salinity because most sequences from *Betaproteobacteria* and *Bacteroidetes* in oligosaline Lake Namco (salinity≈1.2 g L^−1^) on the Tibetan Plateau may be affiliated with typical freshwater clusters (27). Besides climate conditions, this inconsistency may have resulted from the interactive effects of salinity and nutrients (9, 27, 28, 50). In Lake Bosten, the increase in salinity is often coupled with increasing nutrient concentrations due to anthropogenic activities (50). Accordingly, nutrient levels were markedly higher in Lake Bosten (TN=0.94 mg L^−1^) than in Lake Namco (TN=0.28 mg L^−1^), which may have caused the differences observed in BCC between the two lakes. The bacterial community of Lake Bosten was more similar to that of Lake Kelike (TN=0.8 mg L^−1^) than that of Lake Namco, with similar levels of salinity and nutrients, thereby supporting the interactive effect of salinity and nutrients on BCC. Moreover, the high proportion of unclassified *Burkholderiales* and unclassified *Bacteroidetes* in Lake Bosten may be related to their ability to quickly respond to enhanced levels of DOC (23, 24, 33, 35). Although Lake Bosten is mesotrophic, the concentration of DOC (mean value=11 mg L^−1^) is markedly higher than that in oligotrophic Tibetan lakes (4 mg L^−1^) (27, 28) and in eutrophic Lake Taihu (6 mg L^−1^) (48, 49). Our results highlight the selection of differently adapted microorganisms by the environment (53). Detailed comparative investigations of BCC in arid region lakes are still needed to gain robust and deeper insights into the influence of climatic drought and anthropogenic eutrophication on bacterial community and function.

## Conclusions

This study confirmed that the robust seasonal succession of BCC occurred in Lake Bosten, and that water temperature was the key determinant of this succession; vertical variation was not significant. Additionally, only a few bacterial taxa dominated BCC at a certain time, and the dominant taxa shifted markedly on a seasonal time scale. Our results provide strong evidence that the oligosaline and mesotrophic Lake Bosten harbored high proportions of indigenous bacterial taxa, particularly from *Betaproteobacteria* and *Bacteroidetes*, which distinctively differed from typical freshwater clusters. We argue that interactions between salinity and nutrients, reflecting the effects of an arid climate and enhanced anthropogenic activities, respectively, were the main factors resulting in the heterogeneity of BCC between Lake Bosten and freshwater ecosystems. Overall, our results have implications for better understanding of the bacterial responses to environmental changes from freshwater/oligotrophic to oligosaline/mesotrophic conditions in aquatic ecosystems.

## Supplementary Information



## Figures and Tables

**Table 1:** Comparison of bacterial diversities in six clone libraries by means of the Chao1 richness estimator, reciprocal Simpson’s dominance index (RSI), and Shannon’s diversity index (*H′*). Operational taxonomic units (OTUs) were defined at a sequence similarity cut-off of 97%.

Library name	Sample	Clones	OTUs	Chao1 (95% lcl, hcl)	RSI	*H′*	Coverage (%)
AA1	Aug-A-0.5m	81	34	61 (43, 116)	16.4	3.08	75.3
AA2	Aug-A-12m	100	35	112 (59, 282)	11.8	2.96	78.0
AB1	Aug-B-0.5m	94	40	73 (52, 130)	15.4	3.16	73.4
AB2	Aug-B-12m	95	22	35 (25, 173)	7.1	2.34	87.4
MA1	May-A-0.5m	84	18	33 (21, 87)	10.9	2.52	92.9
MA2	May-A-12m	90	26	52 (33, 125)	15.5	2.85	85.6
Total	—	544	98	209 (149, 342)	25.2	3.69	90.8

**Table 2:** Top 10 most abundant (>4.5%) phylotype OTUs in samples from the six bacterial clone libraries

OTU	Clones	Representative OTU (Accession No.)	Closest relatives1) (Accession No.)	Similarity (%)	Source	Division	Percentage of each OTU in each clone library (%)						

							AA1	AA2	AB1	AB2	MA1	MA2	Average
1	51	Aug-B-057 (JQ327385)	*Acinetobacter* sp. N40 (AM778696)	99.8	Lake Negra	*Gammaproteobacteria*	1.2	8.0	16.0	28.4	—	—	9.4
2	46	Aug-B-12-102 (JQ327530)	*Exiguobacterium* sp. H1632 (JF346672)	99.9	coastal sea water	*Firmicutes*	1.2	25.0	2.1	18.9	—	—	8.5
3	31	Aug-A-103 (JQ327235)	Clone SING499 (HM129142)	99.9	Lake Kelike	*Betaproteobacteria*	17.3	9.0	1.1	—	4.8	3.3	5.7
4	30	May-A-011 (JQ327540)	Clone BST7-7 (HQ436594)	99.5	Lake Bosten	*Bacteroidetes*	6.2	3.0	5.3	—	10.7	8.9	5.5
5	30	Aug-B-104 (JQ327423)	*Radiocystis* sp. JJ30-3 (AM710389)	99.9	freshwater reservoir	*Cyanobacteria*	3.7	5.0	18.1	5.3	—	—	5.5
6	29	Aug-B-12-068 (JQ327498)	*Rheinheimera* sp. 193 (JQ012969)	99.8	Baltic Sea	*Gammaproteobacteria*	—	2.0	1.1	13.7	9.5	5.6	5.3
7	28	May-A-091 (JQ327608)	*Limnohabitans* sp. KL6 (HE600678)	99.8	freshwater reservoir	*Betaproteobacteria*	—	1.0	5.3	—	13.1	12.2	5.1
8	27	May-A-062 (JQ327583)	clone SING740 (HM129342)	100	Lake Kelike	*Betaproteobacteria*	6.2	1.0	—	—	10.7	13.3	5.0
9	26	May-A-042 (JQ327567)	clone SING915 (HM129493)	99.5	Lake Kelike	*Bacteroidetes*	—	—	1.1	—	20.2	8.9	4.8
10	25	Aug-A-063 (JQ327210)	clone SING993 (HM129554)	100	Lake Kelike	*Alphaproteobacteria*	14.8	3.0	2.1	1.1	4.8	3.3	4.6

Total	323						50.6	57.0	52.1	67.4	73.8	55.6	59.4

1) Detailed phylogenetic positions of the 10 OTUs can be found in Fig. 6.
