# Supplementary material for: Bacterial Community Composition in Oligosaline Lake Bosten: Low Overlap of Betaproteobacteria and Bacteroidetes with Freshwater Ecosystems
Source: Microbes Environ. 2015 May 15;30(2):180–8. doi: 10.1264/jsme2.ME14177 (PMC4462929; doi:10.1264/jsme2.ME14177)
Supplement: Supplementary file 1 [file 30_180_s1.pdf]

Bacterial community composition in oligosaline Lake Bosten: low overlap of *Betaproteobacteria* and *Bacteroidetes* with freshwater ecosystems

Xiangming Tang[1,*], Guijuan Xie[1], Keqiang Shao[1], Jiangyu Dai[1,2], Yuangao Chen[1], Qiujin Xu[3], Guang Gao[1]

[1] *State Key Laboratory of Lake Science and Environment, Nanjing Institute of Geography and Limnology, Chinese Academy of Sciences, Nanjing 210008, China*

[2] *State Key Laboratory of Hydrology-Water Resources and Hydraulic Engineering, Nanjing Hydraulic Research Institute, Nanjing 210029, China*

[3] *Chinese Research Academy of Environmental Sciences, Beijing 100012, China*

* Corresponding author: xmtang@niglas.ac.cn; Tel. (+86) 25 86882213; Fax (+86) 25 57714759.

Supplemental Materials

Table S1 Typical freshwater clusters previously reported by Crump & Hobbie (8), Eiler & Bertilsson (12), Wu et al. (57), and Zwart et al. (61).

No.	Cluster name	Division	Source
1	LD12	*α-proteobacteria*	Zwart et al. 2002
2	*Brevundimonas intermedia*	*α-proteobacteria*	Zwart et al. 2002
3	CR-FL11	*α-proteobacteria*	Zwart et al. 2002
4	GOBB3-C201	*α-proteobacteria*	Zwart et al. 2002
5	*Novosphingobium subarctica*	*α-proteobacteria*	Zwart et al. 2002
6	LiUU-9-115.2	*α-proteobacteria*	Eiler & Bertilsson 2004
7	A0904	*α-proteobacteria*	Eiler & Bertilsson 2004
8	LiUU-9-283.2	*α-proteobacteria*	Eiler & Bertilsson 2004
9	GKS59	*α-proteobacteria*	Wu et al. 2007
10	alphaI	*α-proteobacteria*	Wu et al. 2007
11	ML-7-85.2	*α-proteobacteria*	Wu et al. 2007
12	*Zymomonas* group	*α-proteobacteria*	Wu et al. 2007
13	*Polynucleobacter necessarius*	*β-proteobacteria*	Zwart et al. 2002
14	LD28	*β-proteobacteria*	Zwart et al. 2002
15	GKS98	*β-proteobacteria*	Zwart et al. 2002
16	*Ralstonia pickettii*	*β-proteobacteria*	Zwart et al. 2002
17	*Rhodoferax* sp. BAL47	*β-proteobacteria*	Zwart et al. 2002
18	GKS16	*β-proteobacteria*	Eiler & Bertilsson 2004
19	*Rhodoferax fermentans*	*β-proteobacteria*	Eiler & Bertilsson 2004
20	LiUU-3-128	*β-proteobacteria*	Eiler & Bertilsson 2004
21	LiUU-5-340.2	*β-proteobacteria*	Eiler & Bertilsson 2004
22	LiUU-5-131	*β-proteobacteria*	Eiler & Bertilsson 2004
23	LiUU-11-179.2	*β-proteobacteria*	Eiler & Bertilsson 2004
24	LiUU-11-174.2	*β-proteobacteria*	Eiler & Bertilsson 2004
25	*Leptothrix*	*β-proteobacteria*	Wu et al. 2007
26	Ellin6095	*β-proteobacteria*	Wu et al. 2007
27	Ellin6067	*β-proteobacteria*	Wu et al. 2007
28	IRD18C08	*β-proteobacteria*	Crump & Hobbie 2005
29	PRD18A09	*β-proteobacteria*	Crump & Hobbie 2005
30	ACK-m1	*Actinobacteria*	Zwart et al. 2002
31	STA2-30	*Actinobacteria*	Zwart et al. 2002
32	MED0-06	*Actinobacteria*	Zwart et al. 2002
33	URK0-14	*Actinobacteria*	Zwart et al. 2002
34	CL500-29	*Actinobacteria*	Zwart et al. 2002
35	CL120-6	*Actinobacteria*	Eiler & Bertilsson 2004
36	acIV-B	*Actinobacteria*	Eiler & Bertilsson 2004
37	ML-5-51.2	*Actinobacteria*	Wu et al. 2007
38	acII-A	*Actinobacteria*	Wu et al. 2007

39	acII-B	*Actinobacteria*	Wu et al. 2007
40	acII-D	*Actinobacteria*	Wu et al. 2007
41	ML-9-87.2	*Actinobacteria*	Wu et al. 2007
42	ML-7-116.2	*Actinobacteria*	Wu et al. 2007
43	ML-9-55.2	*Actinobacteria*	Wu et al. 2007
44	acI-C	*Actinobacteria*	Crump & Hobbie 2005
45	LD2	*Bacteroidetes* (CFB)	Zwart et al. 2002
46	FukuN47	*Bacteroidetes* (CFB)	Zwart et al. 2002
47	PRD01a001B	*Bacteroidetes* (CFB)	Zwart et al. 2002
48	CL500-6	*Bacteroidetes* (CFB)	Zwart et al. 2002
49	GKS2-216	*Bacteroidetes* (CFB)	Zwart et al. 2002
50	cfI	*Bacteroidetes* (CFB)	Wu et al. 2007
51	cfII	*Bacteroidetes* (CFB)	Wu et al. 2007
52	IRD18A11	*Bacteroidetes* (CFB)	Crump & Hobbie 2005
53	IRD18C10	*Bacteroidetes* (CFB)	Crump & Hobbie 2005
54	IRD18C04	*Bacteroidetes* (CFB)	Crump & Hobbie 2005
55	*Methylobacter psychrophilus*	*γ-proteobacteria*	Zwart et al. 2002
56	LiUU-3-334.2	*γ-proteobacteria*	Eiler & Bertilsson 2004
57	CL120-10	*Verrucomicrobia*	Zwart et al. 2002
58	CL0-14	*Verrucomicrobia*	Zwart et al. 2002
59	FukuN18	*Verrucomicrobia*	Zwart et al. 2002
60	Sta2-35	*Verrucomicrobia*	Zwart et al. 2002
61	LD19	*Verrucomicrobia*	Zwart et al. 2002
62	LiUU-11-94	*Verrucomicrobia*	Eiler & Bertilsson 2004
63	LiUU-9-243.2	*Verrucomicrobia*	Eiler & Bertilsson 2004
64	*Synechococcus* 6b	*Cyanobacteria*	Zwart et al. 2002
65	*Planktothrix agardhii*	*Cyanobacteria*	Zwart et al. 2002
66	*Aphanizomenon flos aquae*	*Cyanobacteria*	Zwart et al. 2002
67	*Microcystis*	*Cyanobacteria*	Zwart et al. 2002
68	CL500-11	GNS	Zwart et al. 2002
69	CLO-84	OP10	Zwart et al. 2002
70	CL500-15	*Planctomycetes*	Zwart et al. 2002
71	LiUU-9-218	*Planctomycetes*	Eiler & Bertilsson 2004
72	LiUU-11-47	*Fibrobacteres*	Eiler & Bertilsson 2004

Table S2. Chemical and biological parameters, DGGE bands, and Shannon diversity index (*H'*) for the two sampling stations at different water depths in Lake Bosten. Abbreviations are defined in the text.

Sampling time	station	Depth (m)	TN (mg L^{-1})	NH_4-N (mg L^{-1})	NO_3-N (mg L^{-1})	TP* (μg L^{-1})	Cl^- (mg L^{-1})	SO_4^{2-} (mg L^{-1})	DOC (mg L^{-1})	Chl *a* (μg L^{-1})	Bacterial abundance (10^6 cells mL^{-1})	DGGE bands	*H'*
2010-8-23	A	0.5	1.01	0.09	0.25	0	363	587	7.4	1.88	1.23	28	3.02
2010-8-23	A	4.0	0.89	0.11	0.26	0	385	622	8.6	2.65	2.35	25	2.91
2010-8-23	A	8.0	0.91	0.11	0.24	0	387	624	15.2	4.40	3.86	31	3.18
2010-8-23	A	12.0	1.02	0.11	0.26	0	404	652	9.0	5.99	3.01	27	2.95
2010-8-23	B	0.5	0.92	0.18	0.29	6	378	611	3.9	1.59	1.63	27	3.01
2010-8-23	B	4.0	0.84	0.19	0.29	0	377	611	11.7	2.65	2.25	24	2.91
2010-8-23	B	8.0	0.88	0.19	0.30	9	385	622	6.8	3.34	3.26	23	2.90
2010-8-23	B	12.0	1.00	0.18	0.29	0	442	708	7.6	3.29	2.84	21	2.75
2011-5-10	A	0.5	0.84	0.38	0.39	0	314	501	12.5	2.12	0.57	18	2.51
2011-5-10	A	4.0	0.88	0.32	0.41	0	208	521	11.6	2.00	1.12	20	2.75
2011-5-10	A	8.0	1.06	0.33	0.41	0	427	622	11.5	2.30	1.16	26	2.93
2011-5-10	A	12.0	1.01	0.37	0.43	7	484	714	13.2	4.80	0.49	19	2.58
2011-5-10	B	0.5	0.86	0.41	0.36	0	324	517	12.3	2.21	0.90	26	3.00
2011-5-10	B	4.0	0.96	0.38	0.43	12	430	623	12.6	3.50	0.93	30	3.17
2011-5-10	B	8.0	0.88	0.36	0.43	0	528	778	12.3	3.90	1.28	25	2.99
2011-5-10	B	12.0	1.01	0.32	0.43	10	486	721	13.2	4.30	1.26	34	3.23

*zero means below the detection limit.

Table S3. ∫-Libshuff comparisons of the homology and heterogeneity of the six libraries at different stations and at different water depths in Lake Bosten. Libraries were considered significantly different when the critical P-value <0.0017 (Singleton et al., 2001).

Sampling season	Sample	Comparison	*P*-value	Significantly different
**August**	Aug-A-0.5m vs. Aug-A-12m	XY	0.0502	no
		YX	0.5011	
	Aug-A-0.5m vs. Aug-B-0.5m	XY	0.1951	no
		YX	0.2863	
	Aug-A-12m vs. Aug-B-0.5m	XY	0.3097	no
		YX	0.3016	
	Aug-A-0.5m vs. Aug-B-12m	XY	<0.0001	**yes**
		YX	<0.0001	
	Aug-B-0.5m vs. Aug-B-12m	XY	<0.0001	**yes**
		YX	0.0578	
	Aug-A-12m vs. Aug-B-12m	XY	<0.0001	**yes**
		YX	0.2405	
**August vs. May**	Aug-A-0.5m vs. May-A-0.5m	XY	<0.0001	**yes**
		YX	<0.0001	
	Aug-A-0.5m vs. May-A-12m	XY	<0.0001	**yes**
		YX	<0.0001	
	Aug-A-12m vs. May-A-0.5m	XY	<0.0001	**yes**
		YX	<0.0001	
	Aug-A-12m vs. May-A-12m	XY	<0.0001	**yes**
		YX	<0.0001	
	Aug-B-0.5m vs. May-A-0.5m	XY	<0.0001	**yes**
		YX	<0.0001	
	Aug-B-0.5m vs. May-A-12m	XY	<0.0001	**yes**
		YX	<0.0001	
	Aug-B-12m vs. May-A-0.5m	XY	<0.0001	**yes**
		YX	<0.0001	
	Aug-B-12m vs. May-A-12m	XY	<0.0001	**yes**
		YX	<0.0001	
**May**	May-A-0.5m vs. May-A-12m	XY	0.5914	no
		YX	0.0108	

(A) *α-proteobacteria*

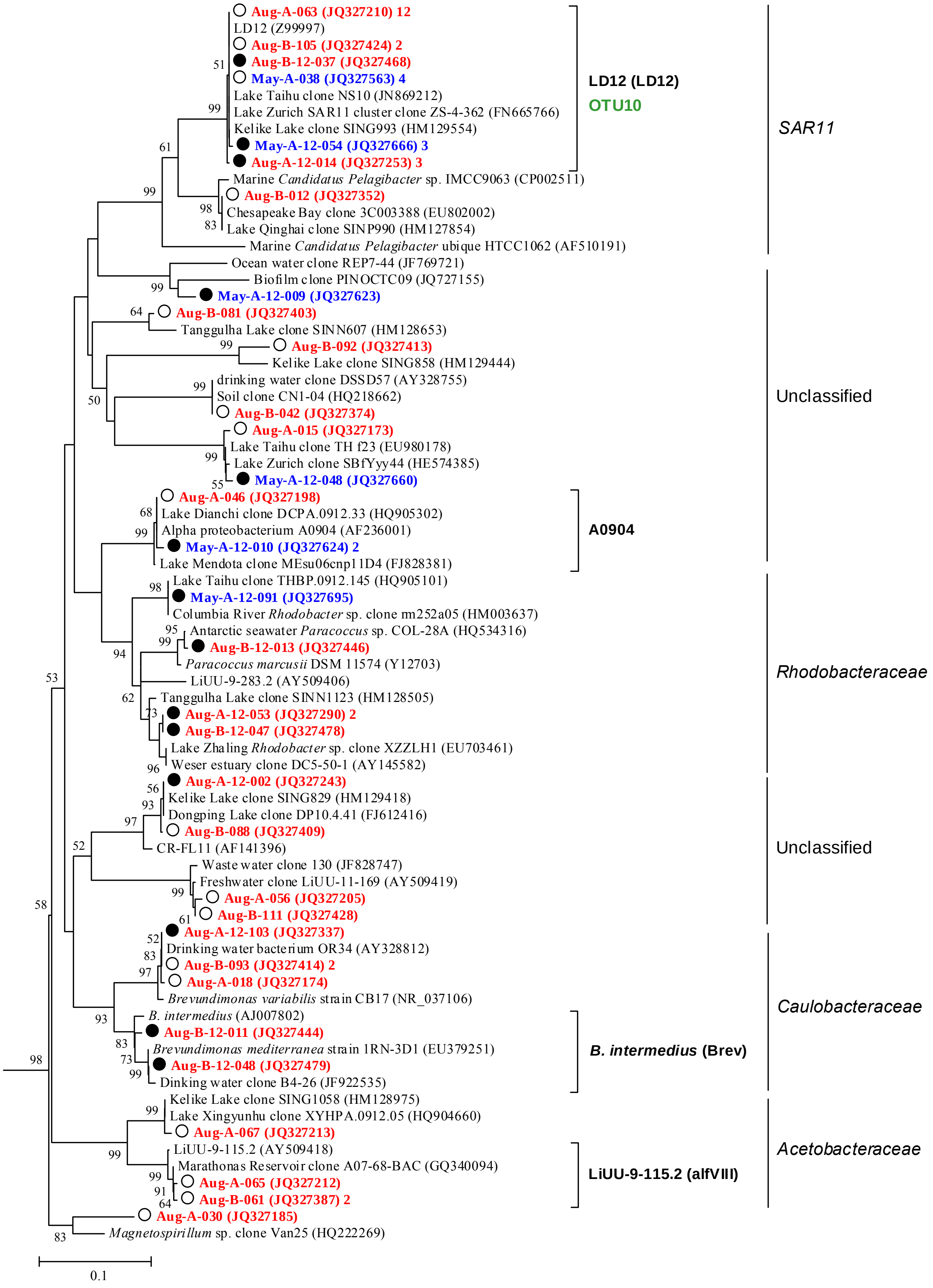

(B) *γ-proteobacteria*

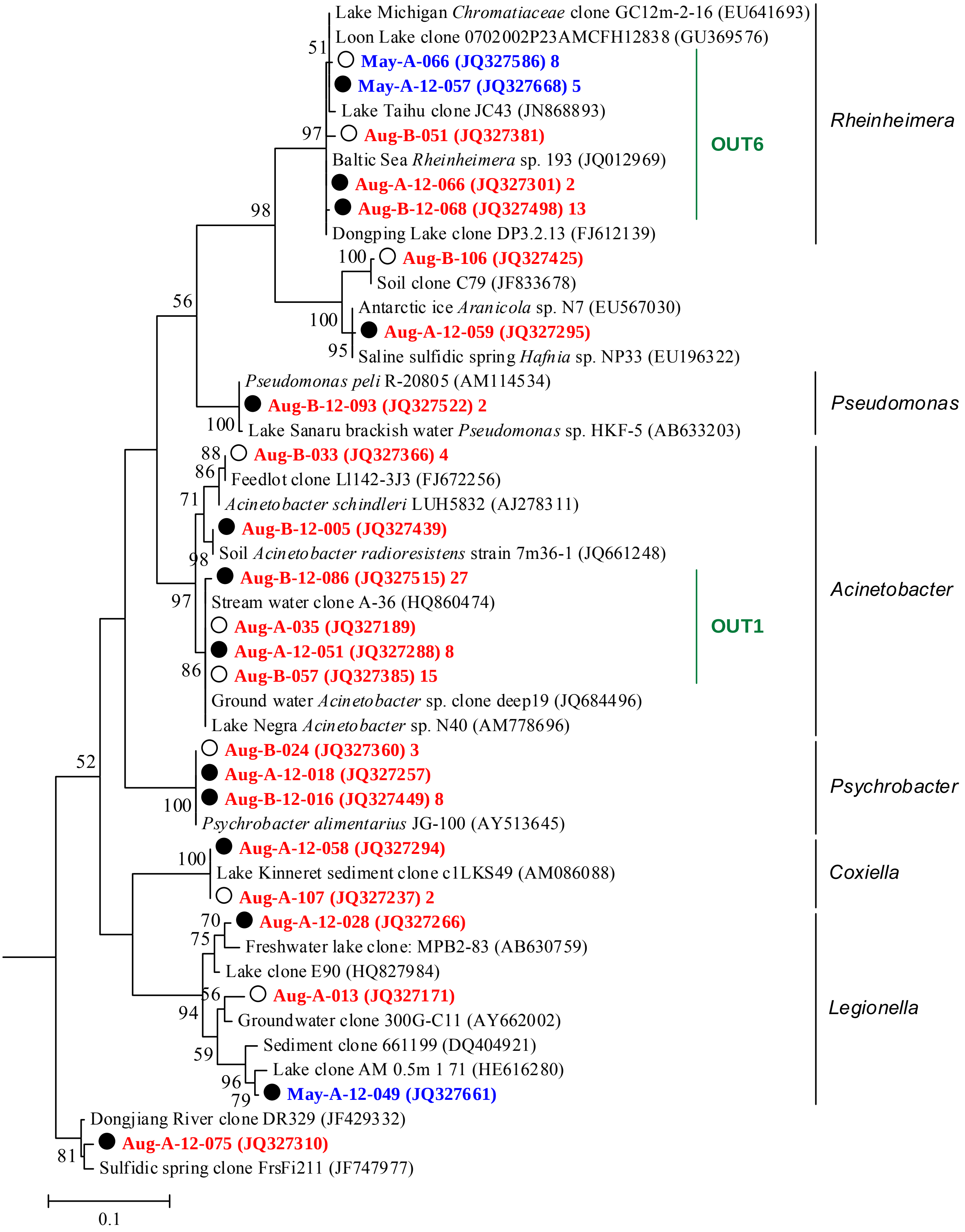

1

(C) *Firmicutes*

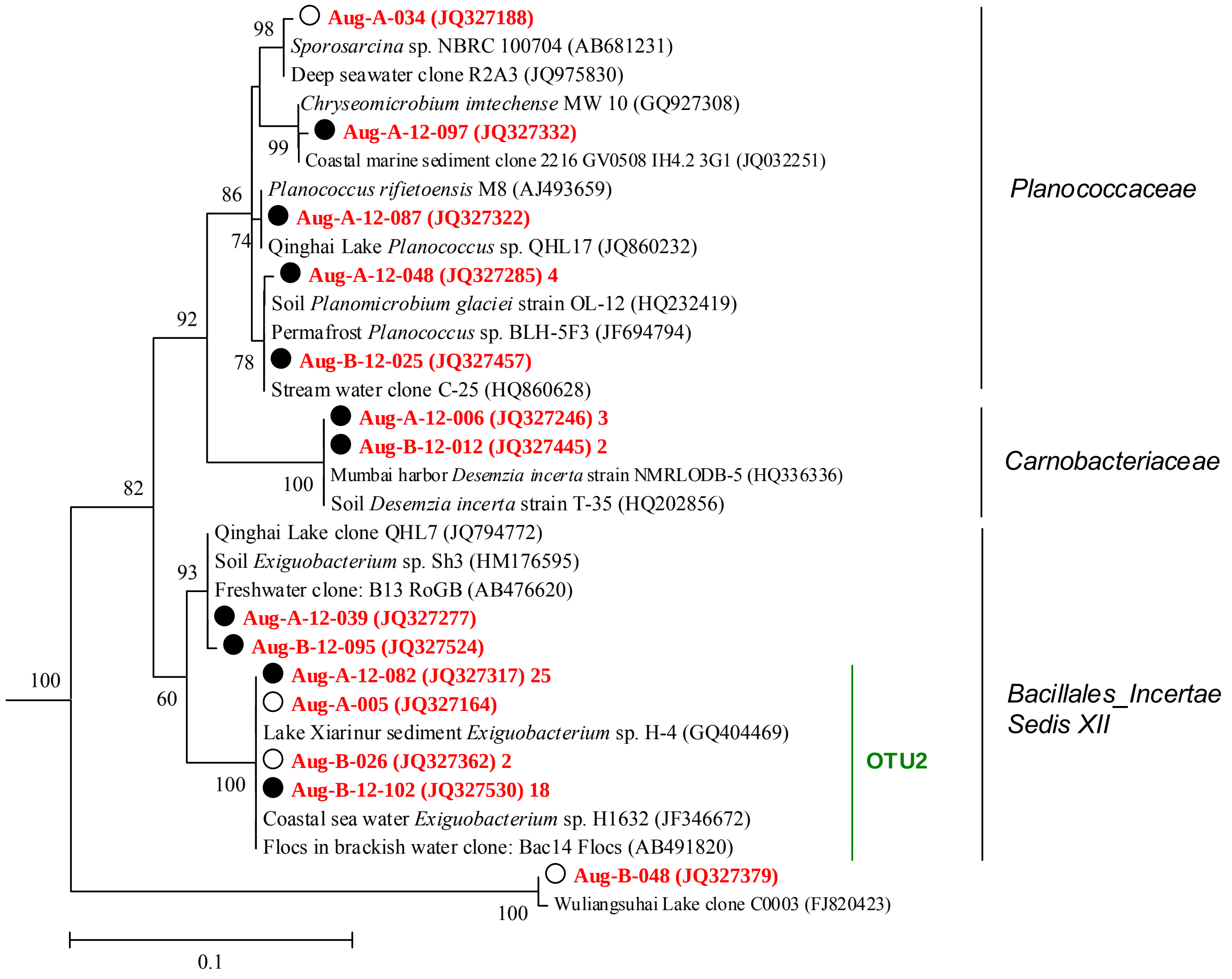

(D) *Cyanobacteria*

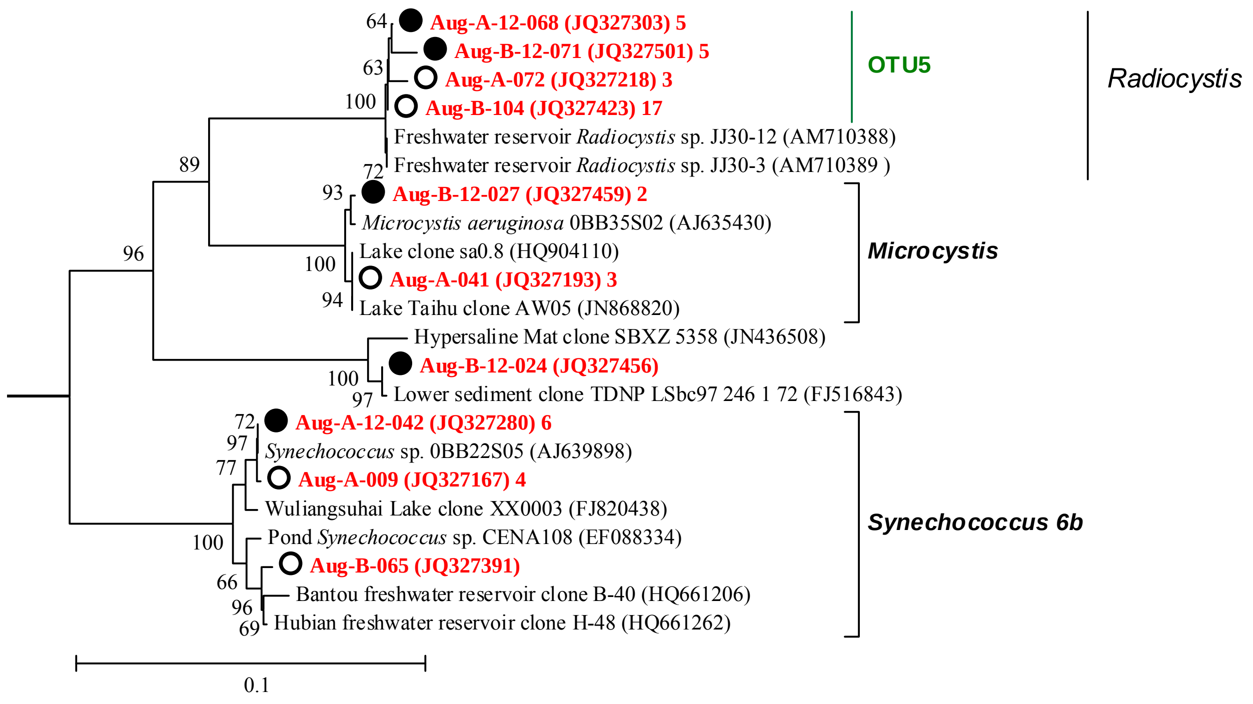

(E) Other phyla

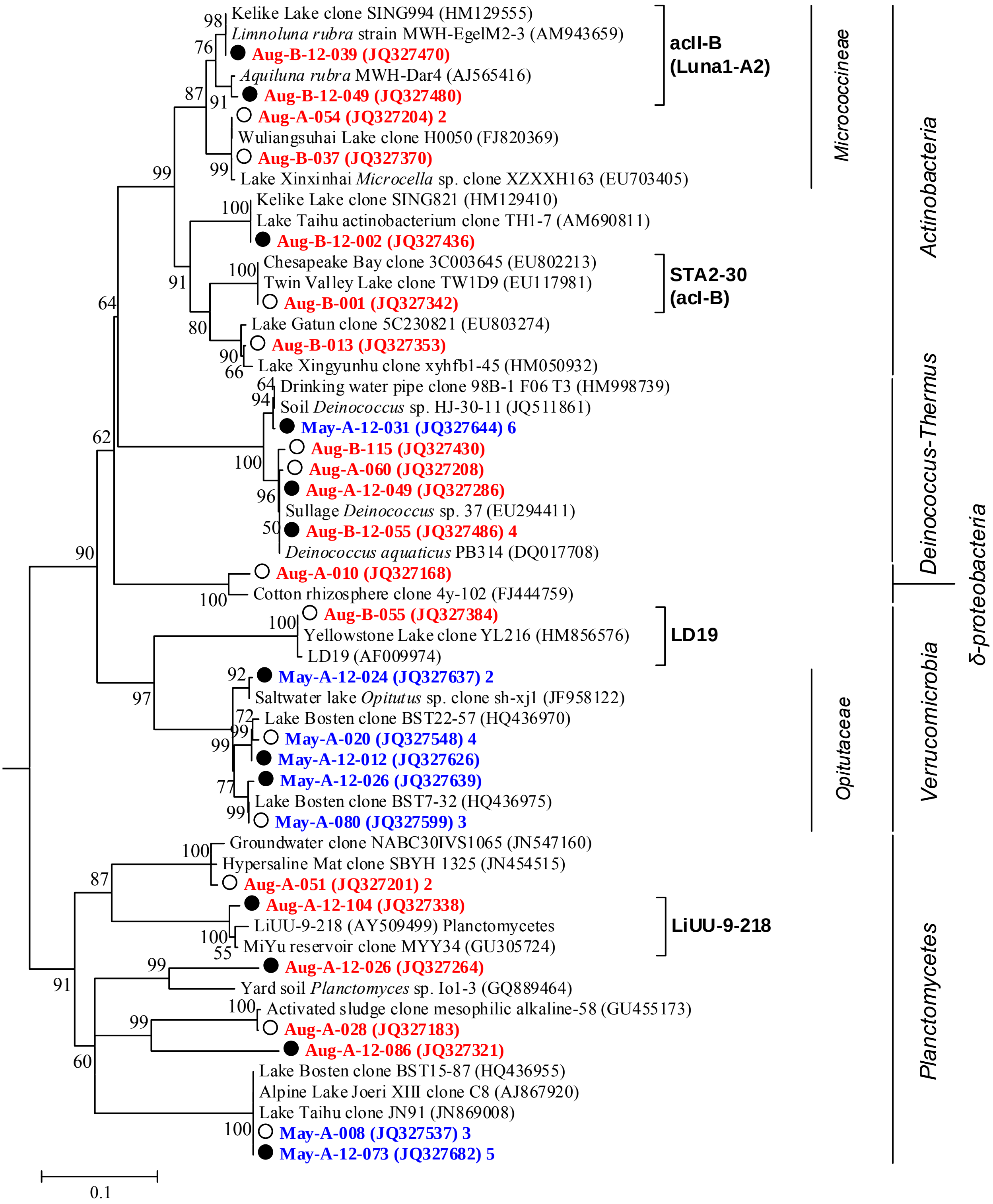

Figure S1 Phylogenetic trees of *α-proteobacteria* (A), *γ-proteobacteria* (B), *Firmicutes* (C), *Cyanobacteria* (D), and other phyla (E) inferred by Maximum Likelihood analysis of partial 16S rRNA gene sequences from six clone libraries in Lake Bosten. A bootstrap test with 1000 replicates was conducted, and only bootstrap values >50% are shown near nodes. Phylogenetic analyses were conducted in MEGA v5.2. Bar: 10% of estimated sequence divergence. Red clones were obtained in August 2010, and blue clones were obtained in May 2011. For each OTU, only one representative clone from each library is shown. The GenBank accession numbers are given in parentheses, followed by the number of clones within each representative clone. The most dominant 10 OTUs (Table 2) in the tree are shown in green. The open circles (○) before the clones represent surface water samples, and the dark filled circles (●) represent bottom water samples. Brackets following clone names indicate typical freshwater clusters previously reported by Crump & Hobbie (8), Eiler & Bertilsson (12), Wu et al. (57), and Zwart et al. (61). Names in brackets following the typical freshwater clusters were tribes or lineages named by Newton and coworkers (34).
